# Implementation and Feasibility of Mechanomyography in Minimally Invasive Spine Surgery

**DOI:** 10.3390/jpm15020042

**Published:** 2025-01-23

**Authors:** Fabian Sommer, Ibrahim Hussain, Noah Willett, Mousa K. Hamad, Chibuikem A. Ikwuegbuenyi, Rodrigo Navarro-Ramirez, Sertac Kirnaz, Lynn McGrath, Jacob Goldberg, Amanda Ng, Catherine Mykolajtchuk, Sam Haber, Vincent Sullivan, Pravesh S. Gadjradj, Roger Härtl

**Affiliations:** Department of Neurological Surgery, Och Spine at New York Presbyterian Hospital, Weill Cornell Medicine, New York, NY 10065, USAnow4003@med.cornell.edu (N.W.); cai4001@med.cornell.edu (C.A.I.); lbm9009@med.cornell.edu (L.M.); jag9177@nyp.edu (J.G.);

**Keywords:** mechanomyography, minimally invasive spine surgery, degenerative spine surgery

## Abstract

**Background**: Mechanomyography (MMG) is a neurodiagnostic technique with a documented ability to evaluate the compression of nerve roots. Its utility in degenerative spine surgery is unknown. **Objective**: To assess the utility of intraoperative MMG during cervical posterior foraminotomy, minimally invasive transforaminal interbody fusion (MIS-TLIF), and tubular lumbar far lateral discectomy. **Methods**: A prospective feasibility study was conducted during which MMG was applied during three procedures. Adhesive accelerometers were placed on two muscle groups per procedure. Stimulus threshold in mA was recorded before and after the decompression of the nerve root. Differences in stimulation thresholds were correlated with operative findings. **Results**: In total, 22 patients were included in this study; 5 patients underwent cervical foraminotomies, 3 underwent MIS-TLIFs, and 14 underwent tubular far lateral discectomies. For the foraminotomies, all cases showed a reduction in stimulation threshold (mean of 3.4 mA) after decompression. For MIS-TLIF cases, there was a limited reduction in the stimulation threshold after decompression (mean 1.7 mA). For far lateral discectomy, there was a mean reduction of 4.3 mA in the stimulation threshold following decompression. **Conclusions**: MMG is a method that may provide intraoperative feedback on the decompression of nerve roots. In the context of MIS-TLIF, MMG showed a limited decrease in stimulus threshold. This may be due to the identification of the nerve occurring after decompression is already underway. For cervical foraminotomies and far lateral discectomies, MMG showed promising results in determining adequate decompression of the nerve root.

## 1. Introduction

Mechanomyography (MMG) is a neurophysiologic modality with a wide range of applications. MMG is a means of mechanically assessing muscle function, often described as a counterpart to electromyography (EMG) [1,2,3]. Non-invasive sensors are placed over the muscle of interest to record and measure contraction [4,5,6,7]. Current applications range from measuring muscle fatigue, intraoperative monitoring devices, and measurements for prosthetic joint replacements [8,9,10,11]. The use of MMG to intraoperatively identify nerves is of particular interest to surgical teams [11]. MMG devices with stimulator probes have been developed to be used in peripheral surgery to avoid iatrogenic nerve injury. Although there are intraoperative devices allowing the implementation of MMG and corresponding literature, MMG is not the standard modality utilized in spine procedures.

The current norm for intraoperative neurophysiologic modalities in spine procedures includes EMG, somatosensory evoked potentials (SSEPs), and motor evoked potentials (MEPs) [12,13,14]. Although there are exceptions at some centers, the typical model of practice includes an intraoperative technologist and remote professional oversight for interpretation [15]. It is important to distinguish between monitoring and mapping when discussing neurophysiologic modalities and techniques. SSEP, EMG, and MEPs are typically used to monitor for adverse events during the procedure, with corrective steps being taken to resolve the events after identification [16]. Mapping involves actively searching for neural tissue or peripheral nerves. In peripheral surgery, this includes localization and determining the extent of damage or compression with triggered EMG [17]. However, electrical noise can pollute the EMG signal.

MMG is an alternative modality that has potential for implementation beyond traditional monitoring. MMG has been used to verify EMG responses when electrical noise is so great that EMG is unreliable [18]. Recent literature has highlighted the utility of MMG in peripheral nerve surgery and quantifying decompression [19]. The authors identified a reduced MMG stimulus threshold when comparing pre- and post-decompression thresholds, possibly identifying an objective measure for determining decompression. In the case of proximal nerve compression, a higher stimulus intensity is needed to elicit a muscle contraction [20]. This physiological phenomenon may be useful in degenerative spine surgery. Intraoperatively identifying the minimum stimulation intensity needed to produce a muscle response both pre- and post-decompression may provide insight into the relative compression of the nerve. This technique could potentially serve as an objective measure of nerve compression during the procedure. This information could help avoid revision interventions by intraoperatively indicating when there may be insufficient decompression. From a medicolegal view, objectifiable decompression would provide clear evidence of intervention [21]. Furthermore, a quantifiable improvement in nerve conduction could reassure both the surgeon and the patient that the operation was technically successful [22].

This could be particularly helpful in surgical procedures with a limited field of view, such as minimally invasive tubular spine surgery or full-endoscopic surgery [23]. This limited view both increases the difficulty of performing the decompression and hampers verification of the extent of decompression. Thus, a quantitative measure of decompression would be extremely advantageous. One study investigated the use of MMG to evaluate nerve decompression during microscopic discectomies after disc herniation and showed promising results [24]. Despite the potential advantages, the technique has not yet found widespread application in spine surgery. Minimally invasive spine surgery is a rapidly evolving field that demands innovation and advanced techniques. For this reason, we conducted a feasibility study to assess the utility of MMG in three procedures to treat degenerative spine diseases. To our knowledge, this is the first study on the topic.

## 2. Materials and Methods

### 2.1. Study Design

We performed a feasibility study utilizing MMG in 3 degenerative spine procedures to determine the extent of intraoperative nerve decompression.

### 2.2. Mechanomyography Technique

**Figure 1:**
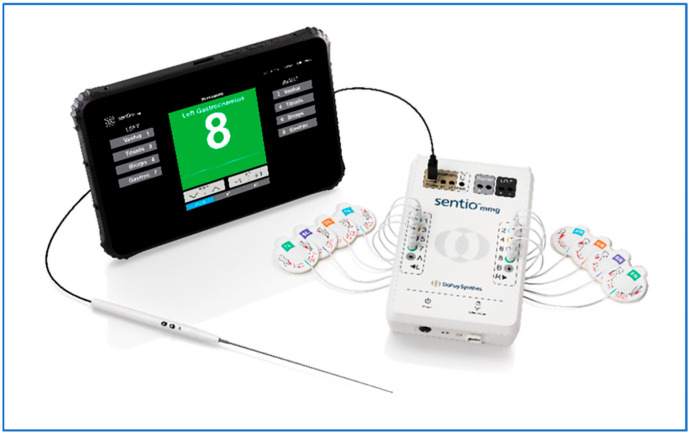
The MMG equipment (DePuy Synthes Sentio, Raynham, MA, USA) consisted of a portable control unit, a portable display, and a sterile monopolar probe.

The MMG equipment (DePuy Synthes Sentio, Raynham, MA, USA) consisted of a portable control unit, a display, and a sterile probe for stimulation (Figure 1). Surface sensors, known as accelerometers, were used to record muscle contraction. Accelerometers are transducers that can measure the acceleration of the body’s surface. Due to the variable innervation of individual muscle groups from spinal nerve roots, all muscles potentially affected during an intervention were equipped with sensors. For interventions of the cervical spine, the trapezius muscle was monitored to cover C3/C4, the deltoid and biceps were monitored to cover C5/C6, and the triceps was monitored to cover C6/C7/C8. For procedures of the lumbar spine, sensors were placed on the vastus medialis to monitor L2/L3/L4, tibialis anterior to monitor L4/L5, biceps femoris to monitor L5/S1/S2, and gastrocnemius to monitor S1/S2. Surgeon-directed stimulation occurred via a sterile monopolar probe. A return electrode was placed lateral to the midline over the latissimus dorsi muscle.

**Figure 2:**
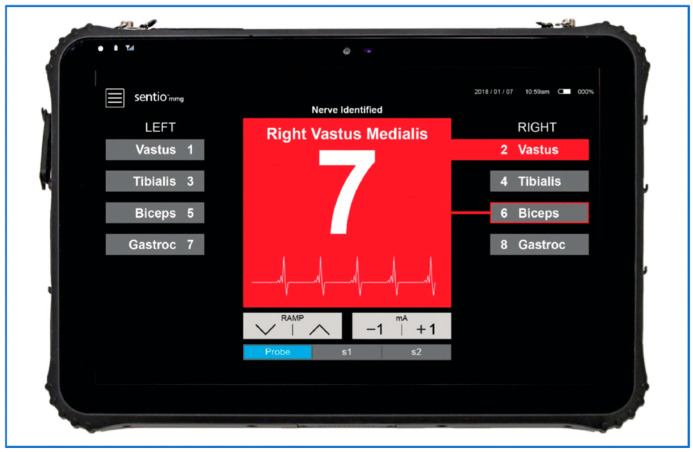
The display shows the strength of the delivered electrical impulse in milliamps (mA), a qualitative representation of the muscle response using color coding, and the MMG waveform at the bottom of the screen.

The MMG response is viewed by the surgeon on the display screen. The display shows stimulation in milliamps (mA), a qualitative representation of muscle activation using color coding, and the MMG waveform at the bottom of the screen (Figure 2). The qualitative representation of muscle activation is coded in red. When there is no muscle activation, it is coded in green. This MMG device was initially developed for peripheral nerve surgery, explaining the somewhat counterintuitive color scheme.

### 2.3. Procedures and Patient Variables

This study examined patients undergoing minimally invasive transforaminal lumbar interbody fusion (MIS-TLIF) [25], minimally invasive (MIS) tubular far lateral discectomy, and cervical foraminotomy. For each procedure, patient-specific data, such as age, sex, BMI, previous procedures, operated level, American Society of Anesthesiology (ASA) score, and duration of symptoms, were recorded. In addition, the following procedure-specific data were documented: duration of surgery, estimated intraoperative blood loss (EBL), length of hospital stay, and procedure-specific complications.

Prior to decompression, the surgeon stimulated the nerve proximal to the site of compression. Stimulation intensity increased in a stepwise manner until the lowest threshold for muscle activation was identified. After satisfactory decompression of the nerve root, the surgeon again identified the lowest threshold for muscle activation. The muscle that elicited a response and the anatomical location of the stimulated nerve were also documented.

## 3. Results

In total, MMG was used for 22 patients (13 men and 9 women). MIS tubular far lateral discectomies accounted for 14 cases, cervical foraminotomy for 5 cases, and MIS-TLIF for 3 cases. There were no reported intraoperative complications during this feasibility study, and no revision surgeries during the short-term postoperative course of 14 days (Table 1).

### 3.1. MIS Tubular Far Lateral Discectomy

**Figure 3:**
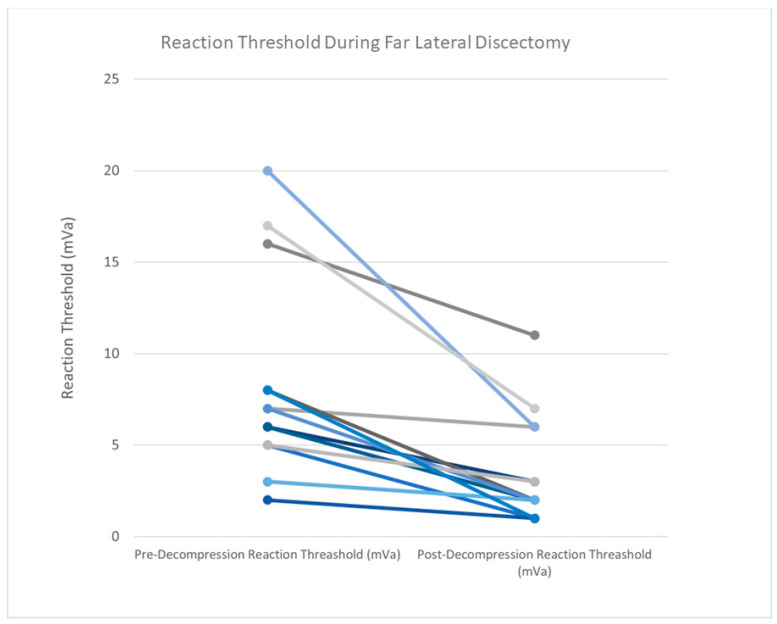
Prior to decompression in far lateral discectomy cases, the MMG stimulus threshold was 7.9 ± 5.6 mA. After the decompression of the nerve, the stimulus threshold was 3.5 ± 2.9 mA. The mean difference in stimulus threshold was 4.4 mA between pre- and post-decompression. Individual lines represent one patient. Colors vary for differentiation.

Procedures were at level L2/3 in one case, level L3/4 in two cases, level L4/5 in three cases, and level L5/S1 in six cases, and two patients had disc herniations at two levels (L4/5/S1 and L2/3 with L5/S1). The mean patient age in this group was 61.3 ± 15.3 years, and the mean BMI was 25.8 ± 4.4. Twelve patients had an ASA score of 2, and one patient had an ASA score of 3. The mean operative time was 120 ± 58.5 min. The EBL was less than 50 mL in 13 cases and less than 250 mL in 1 case. The average hospital stay was 1.4 ± 0.5 days. Prior to decompression, the MMG stimulus threshold was 7.9 ± 5.6 mA. After the decompression of the nerve, the stimulus threshold was 3.5 ± 2.9 mA. The mean difference in stimulus threshold was 4.4 mA between pre- and post-decompression (Figure 3).

### 3.2. Cervical Foraminotomy

**Figure 4:**
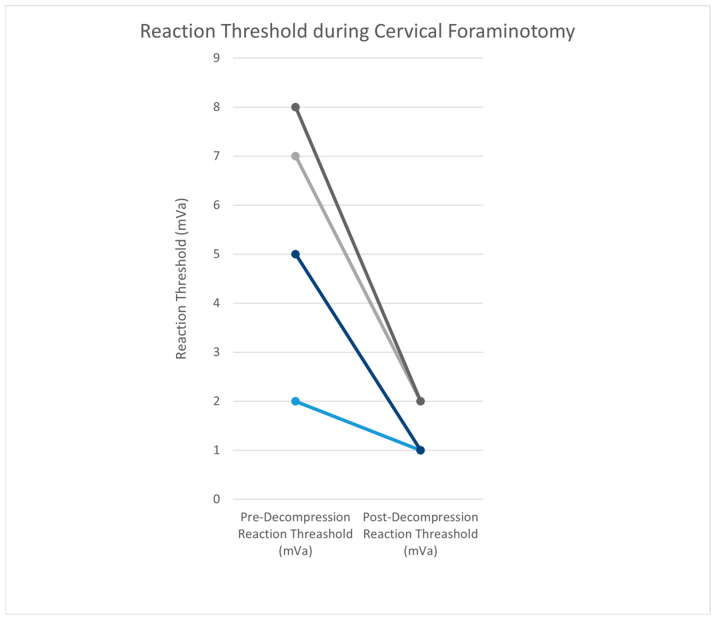
Prior to decompression in cervical foraminotomy cases, the MMG stimulus threshold was 4.8 ± 2.8 mA. After the decompression of the nerve, the stimulus threshold was 1.4 ± 0.5 mA. The mean difference was 3.4 mA between pre- and post-decompression. Individual lines represent one patient. Colors vary for differentiation.

Procedures were at the C6/7 level in all cases, with one case having an additional foraminotomy at the C7/T1 level. The mean patient age in this group was 48.4 ± 17.9 years, and the mean BMI was 23.1 ± 5.2. Four patients had an ASA score of 2, and one patient had an ASA score of 3. The mean operative time of the was 133.4 ± 75.9 min. The EBL was less than 50 mL in four cases and less than 250 mL in one case. The average hospital stay was 1.2 ± 0.4 days. Prior to decompression, the MMG stimulus threshold was 4.8 ± 2.8 mA. After the decompression of the nerve, the stimulus threshold was 1.4 ± 0.5 mA. The mean difference in threshold was 3.4 mA between pre- and post-decompression (Figure 4).

### 3.3. MIS-TLIF

**Figure 5:**
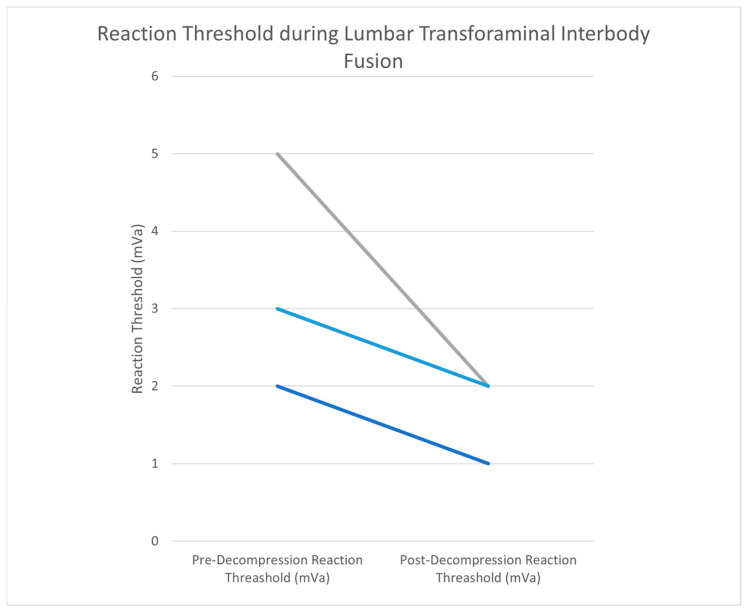
Prior to decompression in MIS-TLIF cases, the MMG stimulus threshold was 3.3 ± 1.5 mA. After the decompression of the nerve, the stimulus threshold was 1.7 ± 0.6 mA. The mean difference was 1.7 mA between pre- and post-decompression. Individual lines represent one patient. Colors vary for differentiation.

Procedures were at the L5/S1 level in one case and at L2/3/4 levels in two cases. The mean patient age was 66.7 ± 4.9 years, and the mean BMI was 25.2 ± 1.1. Two patients had an ASA score of 2, and one patient had an ASA score of 3. The mean procedure time of the TLIF was 232 ± 45 min. The EBL was less than 50 mL in one case, less than 250 mL in one case, and less than 500 mL in one case. The average hospital stay was 3 ± 2 days. Prior to decompression, the MMG stimulus threshold was 3.3 ± 1.5 mA. After the decompression of the nerve, the stimulus threshold was 1.7 ± 0.6 mA. The mean difference in stimulus threshold was 1.7 mA between pre- and post-decompression (Figure 5).

### 3.4. Case Report

**Figure 6:**
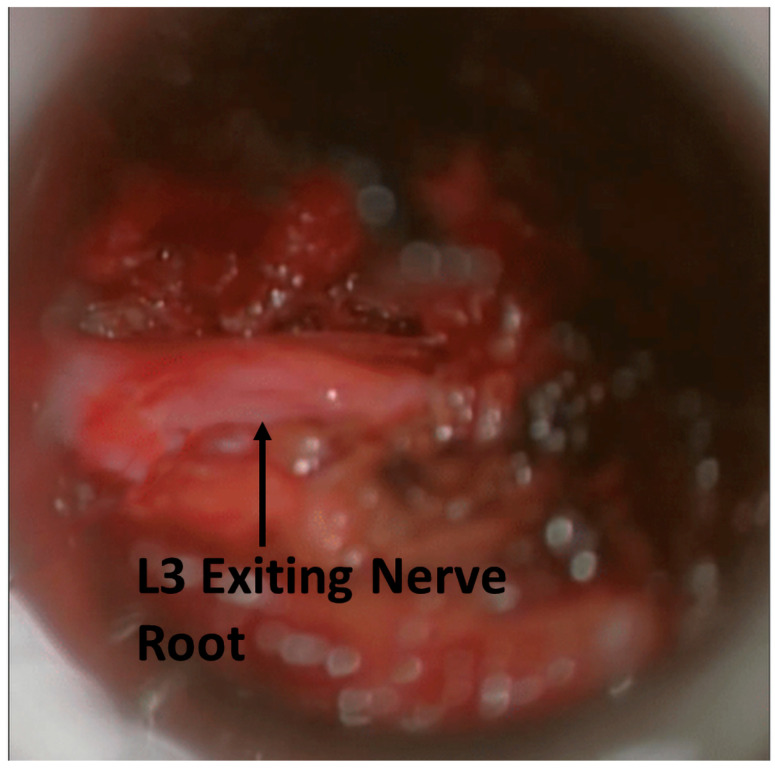
The L3 nerve root was stimulated post-decompression. MMG stimulation threshold did not change. This suggests continued compression of the nerve root.

A 65-year-old female patient presented with severe low back pain, radiating pain to the left buttock, thigh and knee, and left knee extension weakness. Conservative treatment with epidural steroid injections and physical therapy was insufficient in treating her symptoms. MRI imaging showed a left far lateral disc herniation at L3–4. A MIS tubular far lateral discectomy was planned with the use of MMG. The skin incision was made 5 cm off the midline, overlying the L3–4 space. A 15 mm tubular retractor was placed between the transverse process and the pars. The soft tissue was dissected with the use of the microscope. After the identification of the transverse process, the lateral aspect of the facet joint, and the pars, the intertransverse ligament was incised and removed. The pedicle and exiting nerve root were identified. Prior to decompression, the MMG stimulus threshold was greater than 10 mA. After obtaining the stimulus threshold, the disc at L3–4 was incised, and the discectomy was performed. After the exiting nerve root was deemed adequately decompressed, the MMG stimulus threshold did not change (Figure 6). It was decided to further explore the disc space caudally, and an additional disc fragment was identified and removed. After removal, the MMG stimulus threshold decreased from greater than 10 mA to 3 mA. After surgery, the patient was observed for 3 h in recovery and demonstrated adequate mobilization without pain. The patient was safely discharged home on the same day.

## 4. Discussion

### 4.1. MIS Tubular Far Lateral Discectomy

MMG in far lateral discectomies had the largest mean decrease in stimulus threshold (4.3 mA) of the three procedures. During far lateral discectomies, the spinal nerve of the affected segment can be reached early in the surgical course via the dorsal tubular approach. This allows stimulus threshold testing using MMG prior to any decompressive steps being taken. The first stimulus threshold is a true baseline intensity. The added benefit of MMG testing is apparent in MIS cases. The narrow tubular retractor restricts the view of the operating field. This can lead to missed disc fragments that are compressing the nerve outside the visual field. The previously described case report illustrates this point. MMG may be used to confirm adequate decompression in these cases.

### 4.2. Cervical Foraminotomy

The mean MMG stimulus threshold in cervical foraminotomies decreased by 3.4 mA post-decompression. There was less than a 1 mA difference between mean decreases when compared to the far lateral cohort (4.3 mA). This difference may be attributed to the differences in cohort size. Again, the nerve can be identified, and the stimulus threshold obtained prior to any decompressive work being performed. This pre-decompression baseline may allow MMG to be utilized as a tool to confirm adequate decompression.

### 4.3. MIS-TLIF

When compared to the other two cohorts, the mean difference in the stimulus threshold between pre- and post-decompression (1.7 mA) was the least for MIS-TLIF. This minimal difference between pre- and post-decompression may be due to when the nerve is accessible relative to decompression. The facet joint is removed prior to accessing the nerve and before MMG threshold testing. Facetectomies are commonly used as a decompressive maneuver [25]. Decompression of the nerve root occurs before MMG testing so a true pre-decompression baseline is not established in MIS-TLIF procedures. This may contribute to the relatively small decrease in stimulation threshold. The procedural steps and approach of a TLIF may prevent MMG from having value as an objective measure of decompression.

**Figure 7:**
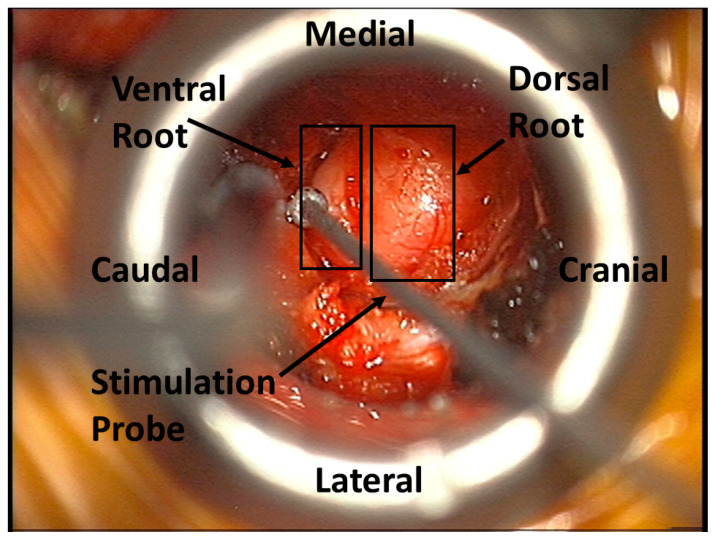
The MMG stimulation probe identifying the ventral nerve root during a cervical foraminotomy.

Care must be taken when determining the site of nerve stimulation. If stimulation is occurring proximally to the dorsal root ganglia, the ventral and dorsal roots may both be attractive stimulation sites. This is illustrated in Figure 7 during a cervical foraminotomy. Given that MMG is measuring a motor response, the identification and stimulation of the ventral root are paramount. Stimulation of the dorsal root may result in absent or confounding information.

As reported by Holland et al., the stimulus thresholds prior to decompression were greater than the thresholds after decompression [20]. This may suggest nerve compression and subsequent alleviation post-decompression. Wessell et al. discussed the importance of obtaining lower intrapatient stimulus thresholds when using MMG testing [24]. Our data agree with this suggestion, as all patients obtained lower stimulus thresholds post-decompression, but absolute thresholds varied among patients.

Our feasibility study demonstrated the reproducibility and potential advantages of using MMG in far lateral discectomies and cervical foraminotomies to treat disc herniation and foraminal stenosis. The use of MMG to verify nerve decompression in herniated discs has been previously reported in the literature [11]. A positive correlation between decreased stimulation threshold and postoperative improvement in VAS scores was reported in a cohort of 46 patients using MMG in lumbar decompression surgery [16]. A reduction in stimulation threshold is not confined to MMG. A study examining electromyography stimulation thresholds before and after lumbar discectomies found an average difference of 4.4 ± 4.0 mA [26]. Additionally, they describe two cases in which the stimulus threshold did not change, and on further exploration, they found other sites of compression not previously identified.

Future implementation of this MMG technique may provide the surgeon with an objective measure of nerve decompression providing valuable feedback on the adequacy of surgical maneuvers. Verifying decompression is especially important in MIS cases, as the surgical field is limited. MMG could potentially reduce operative time and iatrogenic risk by explicitly informing the surgeon when decompression is complete. The surgeon-driven method described in this study eliminates the need for an intraoperative neurophysiology specialist, reducing patient costs and hospital staffing requirements. However, an intraoperative neurophysiology specialist would still be required if traditional modalities such as SSEPs and MEPs are desired. While implementation of this technique may require initial training on the setup and surgical application, the potential benefits outweigh the learning curve.

### 4.4. Limitations

Minimal differences in stimulation threshold for MMG were observed during application in MIS-TLIFs, as previously described. The placement of sensors over muscle groups must be completed by knowledgeable providers to avoid monitoring incorrect innervation patterns. The simultaneous use of MMG and traditional neuromonitoring requires close communication between all providers to reduce potential false positive alerts. Neuromuscular blockers prevent MMG stimulation threshold testing requiring case-specific consideration if muscle relaxation is desired. For cases that utilize MMG, no or short-acting neuromuscular blockers should be utilized. If long-acting neuromuscular blockers are given, a reversal agent may be needed. Regardless of the neuromuscular blocker used, a train-of-four should be run to ensure clearance prior to MMG testing. Due to the design of the study and the small sample size, descriptive statistics were applied. Descriptive analysis is limited in application, but more in-depth analyses would have a high risk of bias.

## 5. Conclusions

MMG is a straightforward method that may provide intraoperative feedback on the relative decompression of nerve roots. Of the three procedures examined in this study, MMG was most valuable in MIS tubular far lateral discectomies and cervical foraminotomies. In the context of MIS-TLIF, MMG was of limited value in our study. MMG was easy to implement and readily available for desired procedures. At this time, importance should be placed on obtaining relatively lower thresholds post-decompression. Follow-up studies with large cohorts are needed to identify relative and absolute stimulation thresholds and their relationship with the extent of decompression and postoperative outcomes.

## Figures and Tables

**Table 1:** Patient demographics and operative factors.

Characteristic	Frequency (*n* = 22)
Sex	
Male	13 (59.1%)
Female	9 (40.9%)
Procedure	
MIS Tubular Far Lateral Discectomies	14 (63.6%)
Age (years)	61.3 ± 15.3
BMI (kg/m^2^)	25.8 ± 4.4
Blood Loss (*n*, %)	
<50 mL	13 (92.8%)
<250 mL	1 (7.2%)
Mean Operative Time (min)	120 ± 58.5
Mean Hospital Stay (days)	1.4 ± 0.5
Cervical Foraminotomy	5 (22.7%)
Age (years)	48.4 ± 17.9
BMI (kg/m^2^)	23.1 ± 5.2
Blood Loss (*n*, %)	
<50 mL	4 (80%)
<250 mL	1 (20%)
Mean Operative Time (min)	133.4 ± 75.9
Mean Hospital Stay (days)	1.2 ± 0.4
MIS-TLIF	3 (13.6%)
Age (years)	66.7 ± 4.9
BMI (kg/m^2^)	25.2 ± 1.1
Blood Loss (*n*, %)	
<50 mL	1 (33.3%)
<250 mL	1 (33.3%)
<500 mL	1 (33.3%)
Mean Operative Time (min)	232 ± 45
Mean Hospital Stay (days)	3 ± 2

## Data Availability

The data presented here are available upon request from the corresponding author.
